# Differences in the longitudinal change of behaviours related to dementia in long-term care: a growth mixture modelling approach

**DOI:** 10.1186/s12877-023-03933-y

**Published:** 2023-04-28

**Authors:** Annie Robitaille, Linda Garcia, Graciela Muniz Terrera

**Affiliations:** 1 Faculty of Health Sciences, University of Ottawa, Ottawa, ON Canada; 2 Centre of Excellence, Perley Health, Ottawa, ON Canada; 3 Ohio University Heritage College of Osteopathic Medicine, Ohio University, Athens, OH USA; 4 Edinburgh Dementia Prevention, University of Edinburgh, Edinburgh, UK

**Keywords:** Dementia, Responsive behaviours, Long-term care, Longitudinal data, Growth mixture model

## Abstract

**Background:**

There is still a need for more information about the different trajectories of responsive behaviours that people living with dementia present in long-term care homes (LTC). Objective. This study identified subgroups of individuals with similar trajectories of responsive behaviours related to dementia in LTC and evaluated the role of demographic variables, depressive symptomatology, social engagement, cognitive functioning, and activities of daily living (ADL) on class membership.

**Methods:**

Growth mixture models were run using data from the Continuing Care Reporting System.

**Results:**

Results suggest that change in responsive behaviours is best represented by seven classes of trajectories. The largest class was composed of individuals who presented the lowest frequency of behaviours upon entry in LTC that increased at a slow linear rate. The other classes were composed of individuals who presented different frequencies of behaviours upon entry in LTC and varying rates of change (e.g., individuals who presented a low frequency of behaviours upon entry in LTC that increased at a linear rate followed by a decrease in the later months, individuals who presented a high frequency of responsive behaviours upon entry in LTC and that remained stable). Cognitive functioning, social engagement, depressive symptomatology, and ADL were markers of class membership.

**Conclusions:**

These findings can help identify individuals at increased risk of presenting a high frequency of responsive behaviours and highlight interventions that could decrease behaviours in LTC.

## Introduction

As the population is increasing rapidly in age, the number of people with dementia is also increasing and is expected to triple by 2050 [1], making dementia a global challenge requiring worldwide attention. As stressed by the Lancet Commission on Dementia Prevention, Intervention, and Care, although a cure for dementia has not yet been found, several actions can be taken to significantly modify the dementia-related trajectories (e.g. delayed onset of dementia) and considerably improve the lives of those living with dementia [2] and their care partners [3].

In addition to the decline in cognitive abilities and activities of daily living (ADL) seen in people living with dementia, behavioural changes are also common [4, 5]. These include behaviours such as appearing frustrated and restless, striking another person when asked to undress, and behaviours deemed socially inappropriate. These behaviours have been termed differently by different researchers in the literature such as “agitation” [6, 7], “aggressive, agitated, or disruptive (AAD) behaviours” [8], disruptive or inappropriate behaviour [9], challenging behaviour [10], need-driven behaviours [11], and responsive behaviours [12]. In line with the importance of words used when talking about dementia, the term “responsive behaviours” will be used for this paper [13, 14].

Caring for a person living with dementia who presents responsive behaviours can be more difficult and time-consuming than caring for a person living with dementia that does not present any of these behaviours [2, 15, 16]. Given that over 60% of people in long-term care (LTC) have dementia [17] and that the prevalence of behaviours related to dementia is more frequent in LTC homes [2]; caregivers (i.e. family members and LTC staff) of people living with dementia in these settings are at an increased risk of stress-related psychosocial and physical health effects [15, 18, 19].

A better understanding of whether and how behaviours related to dementia are likely to be expressed in LTC continues to be an area of great need. Much of the research to date has been cross-sectional, examining the relationship between responsive behaviours related to dementia at one point in time rather than examining the trajectory of these behaviours in LTC [20–22]. Examining the trajectory of these behaviours upon entry into LTC and potential predictors (e.g., age at entry, level of social engagement) of their paths can shed some light on changes that occur in LTC as well as possible risk factors for presenting responsive behaviours. In a previous study, we examined the trajectory of dementia-related behaviours in LTC and found that behaviours related to dementia tend to increase upon entry in LTC and subtly level off at later assessment times (i.e., negative quadratic trend). We also found that younger males with higher depressive symptomatology, more impairment with ADL, and less social engagement tended to present more responsive behaviours [23]. However, this approach assumes that behavioural changes in LTC can be described by a single trajectory. It is more reasonable to assume that there might be heterogeneity in the paths and that certain profiles might cluster together, allowing us to demonstrate the development of different patterns. For example, it is likely that some individuals enter LTC exhibiting few behaviours but show a fast increase in behaviours, whereas others might enter LTC exhibiting few behaviours with no further increase over time.

Developments in the analysis of longitudinal data have resulted in an ability to better focus on understanding heterogeneity in trajectories. One such development referred to as Growth Mixture Modeling (GMM) allows for the identification of different classes of individuals whose trajectories show clusters of similar patterns rather than describing a homogenous trajectory within a given population [24]. GMM provides information about the optimal number of classes and characteristics of each class including the average trajectory, proportion of the membership, and predictors of class membership.

This approach will extend our previous research on the topic and will provide further insight into how to better manage responsive behaviours [23]. The importance of key factors such as social engagement, depressive symptomatology, and activities of daily living (ADL) could be examined more closely in terms of whether they have an impact on class-specific trajectories of responsive behaviours but also on whether these factors help to discriminate individuals’ membership to the different classes. For example, individuals who are more socially engaged when entering LTC may be more likely to be in the class with a low frequency of responsive behaviours. The information gained from a better understanding of the different trajectories of responsive behaviours in LTC and factors associated with class allocation will generate knowledge that will permit the tailoring of interventions and lead to more person-centred action plans.

The purpose of this study was to (1) identify subgroups of persons living with dementia describing the longitudinal change in dementia-related responsive behaviours, and (2) evaluate the role of demographic variables, depressive symptomatology, social engagement, cognitive functioning, and ADL on differences in class membership.

## Methods

### Data

The following section describes the same methods that were used in our previous work [23]. For further information about the methods see [23]. Data developed by InterRAI (http://www.interrai.org) was used for this study [25, 26]. More specifically, the Minimum Data Set (MDS) component of InterRAI’s Resident Assessment Instrument (RAI) was used. In Canada, it is also referred to as the Continuing Care Reporting System (CCRS) (https://www.cihi.ca/en/continuing-care-metadata). The CCRS was implemented across LTC homes in the province of Ontario in 2003–2004. It was subsequently partially or completely implemented across eight additional Canadian provinces/territories. Only data from Ontario was used for the current study, given that more participants are included and for longer periods as compared to other provinces [23]. All publicly funded LTC homes are mandated to submit MDS-CCRS data to CIHI quarterly. The questionnaires include demographic, functional, and clinical information about the residents (e.g., Cognitive function, Activity patterns, Physical function, and Psychosocial well-being). The questionnaires are completed by trained nurses every three months [23].

### Measures

#### Responsive behaviours

Responsive behaviours were assessed using the MDS Aggressive Behaviour Scale (ABS) [27]. The ABS assesses the frequency and intensity of residents’ behaviours in the last 7 days. The ABS includes the following 4 items: resisting care, physical abuse, socially inappropriate behaviour, and verbal abuse. Items range from 0 (behaviour not exhibited in last 7 days) to 3 (behaviour occurred daily) with total scores ranging from 0 to 12; with higher scores representing a greater number and intensity of behaviours. Good validity and reliability have been reported for the MDS ABS in LTC [27, 28]. The ABS is completed by a trained nurse.

#### Covariates

We included gender (men = 0; women = 1), and age at entry in LTC as covariates. We centred age on its mean to improve the interpretation of results [23].

#### Cognitive functioning

The Cognitive Performance Scale (CPS) was used to assess residents’ levels of cognitive functioning [29]. The CPS includes 5 items with total scores ranging from 0 (no cognitive impairment) to 6 (very severe impairment); with higher scores indicating more cognitive impairment. Residents are assessed on whether or not they are comatose, as well as their abilities related to short-term memory, cognitive skills for daily decision-making, and eating in the past 7 days [23]. Good validity and reliability have been reported for the MDS CPS in LTC [28, 30, 31]. The CPS is completed by a trained nurse.

#### Depressive symptomatology

The Depression Rating Scale (DRS) [32] was used to assess depressive symptomatology. The total DRS score is composed of 7 items (e.g., “Sad, pained, worried facial expression”). Each item is rated on a 3-point scale ranging from 0 (not present in the past 30 days) to 2 (present 6 or 7 days per week in the past 30 days). Total scores range from 0 (no mood symptoms) to 14 (all mood symptoms present). Good validity and reliability have been reported for the DRS in LTC [28, 32]. The DRS is completed by a trained nurse.

#### Social engagement

Social engagement was assessed using the Index of Social Engagement (ISE) [33]. Residents are assessed on whether they engaged in 6 different social engagement behaviours (e.g., accepting invitations into most group activities) in the previous 7 days with 0 = no and 1 = yes. The total ISE score ranges from 0 to 6 with higher scores indicating more social engagement. Good validity and reliability have been reported for the ISE in LTC [33]. The ISE is completed by a trained nurse.

#### Activities of Daily Living (ADL)

ADL was assessed using section G of the MDS (i.e., Physical Functioning and Structural Problems) Activities of Daily Living–Long Form. Residents are assessed during the past 7 days on 7 activities (e.g., bed mobility, eating, and dressing). Scores range from 0 (Independent) to 4 (total dependence) with the total score ranging from 0 to 28; with higher scores indicating more impairment. The ADL is completed by a trained nurse.

### Sample

We used data from assessments that were completed between 2006 and 2012 and selected only older adults identified as living with “Alzheimer’s disease” or “dementia other than Alzheimer’s disease” for inclusion in the sample. This diagnosis is completed by a trained nurse using their clinical judgement, feedback from other staff members, communication with the residents and care partners, observation of the person, and review of charts and other secondary documents. The high sensitivities and specificities of the interRAI assessment compared to administrative records provide support for the accuracy of diagnosis of dementia [34].

Out of the 17,112 older adults living with dementia, we only selected those 65 years of age and older, resulting in the exclusion of 272 cases. We further excluded 30 individuals because they were missing data on the predictor variables. The final sample included 16,810 older adults ranging in age from 65 to 109 [23]. Persons living with dementia received between 1 and 24 assessments with a mean of 6 assessments (SD = 4.16). We only included the first 11 assessment times given the low number of cases with data after 11 assessments [23]. See Table 1 for means and standard deviations for responsive behaviours at the initial assessment and the number of assessments on each occasion. See Table 2 for descriptive statistics for the initial assessment in LTC.

**Table 1:** Descriptive statistics for responsive behaviours and the number of assessments

	Sample	Responsive Behaviours
	N (percentage of data present)	Mean(SD)
Initial assessment	16,804(100)	1.67(2.51)
3 months	14,266(84.9)	1.88(2.66)
6 months	12,284(73.1)	1.97(2.72)
9 months	10,788(64.2)	2.03(2.71)
12 months	9309(55.4)	2.07(2.71)
15 months	7898(47.0)	2.12(2.73)
18 months	6688(39.8)	2.20(2.76)
21 months	5713(34.0)	2.25(2.78)
24 months	4772(28.4)	2.32(2.83)
27 months	3831(22.8)	2.35(2.81)
30 months	2840(16.9)	2.40(2.88)

The percentage of data present is from the baseline

*SD* Standard deviation

**Table 2:** Descriptive statistics at initial assessment in LTC and reasons for discharge

Variables	N	%
Gender		
Men	4782	28.5
Women	12,022	71.5
Marital status		
Married	5485	32.6
Widowed	9686	57.6
Divorced/Separated/ Never married	1321	7.8
Education		
Less than high school	5707	33.9
High school	2891	17.2
Technical/trade	900	5.4
Some college	922	5.5
University	1157	6.9
Missing	5227	31
Reason for discharge		
Deceased	4256	25.3
Hospital	3963	23.5
Residential care	1095	6.5
Private home	871	5.2
Other or Unknown Discharge Disposition	6619	39.4
	Mean	SD
Age	84.89	6.6
Depression	2.00	2.3
Social Engagement	2.78	1.8
Activities of Daily Living	13.26	7.9
Cognitive impairment	3.13	1.4

*n* = 16,804

*SD* Standard deviation

See Table 1 for baseline data on responsive behaviours

### Statistical analyses

Growth mixture models were run to identify unobserved groups of individuals with similar trajectories of responsive behaviours related to dementia. GMM provides estimates of the average responsive behaviour trajectories and variation for older adults in each latent class. Based on our previous publication where we reported that changes in people living with dementia follow a non-linear trajectory, we fitted a GMM that estimated curvilinear trajectories for each class.

The number of classes is unknown a priori; therefore, models were fit with an increasing number of classes (one class, two classes, three classes, and four classes). To decide on the number of classes, Bayesian Information Criteria (BIC) values were compared from each model. Models with lower BIC values were considered better fitting. The use of BIC values has been supported in the literature [35, 36] with a BIC value difference of 10 or more indicating a better model [37]. In addition, classification quality using entropy values (ranges from 0 to 1 with higher values meaning that individuals are better discriminated between classes) [38], Lo–Mendell–Rubin (LMR) likelihood ratio test, bootstrapped likelihood ratio test (BLRT), and interpretability of classes was considered [36]. A significant LMR and BLRT test suggests that the model with k classes has a better fit than the same model with k-1 classes [36]. Mplus version 7.2 was used to run the GMMs [39, 40]. The model was estimated by maximum likelihood with robust standard errors and missing data assumed to be MAR.

Social engagement, cognitive functioning, depressive symptomatology, and ADL at baseline were added as covariates of class membership. In addition, baseline age, gender, education, social engagement, cognitive functioning, depressive symptomatology, and ADL were included to examine their association with the intercept and rate of change of each of the identified groups of trajectories (latent classes).

## Results

### Number of classes

We selected a seven-class model of responsive behaviours, given its lower BIC values, good classification of individuals in each class, the significant BLRT values when compared to the six-class model, and interpretability of the classes. See Table 3 for the BIC, entropy, LMR, and BLRT values for models from two to eight classes.

**Table 3:** BIC, entropy, LMR, and BLRT values for all models

	2 classes	3 classes	4 classes	5 classes	6 classes	7 classes	8 classes
BIC	385,931.18	382,114.27	380,608.55	379,533.21	378,595.49	377,863.76	377,456.75
AIC	385,536.99	381,519.11	379,812.43	378,536.12	377,397.44	376,464.75	375,856.77
Entropy	.92	.88	.87	.85	.84	.82	.82
LMRp	< .001	< .001	.04	.49	.75	.24	.16
BLRTp	< .001	< .001	< .001	< .001	< .001	< .001	.15

*BIC* Bayesian information criterion, *AIC* Akaike information criterion, *LMRp p*-value from the Lo–Mendell–Rubin likelihood ratio test, *BLRTp* Bootstrapped likelihood ratio test

### Responsive behaviour trajectories

#### Class 1

The largest class was composed of 11,601 individuals (69% of the sample) who presented the lowest frequency of responsive behaviours upon entry in LTC (0.72; SE = 0.02) and that increased at a slow linear rate of 0.09 (SE = 0.02). See Table 4 for all estimates for the 7-class growth mixture model of behaviours related to dementia.

**Table 4:** Estimates for the 7-class growth mixture model of behaviours related to dementia

	Class 1 (*N* = 11,601, 69%)	Class 2 (*N* = 2220, 13%)	Class 3(*N* = 917, 5%)	Class 4 (*N* = 916, 5%)	Class 5 (*N* = 485, 3%)	Class 6 (*N* = 360, 2%)	Class 7 (*N* = 305, 2%)
	Estimates (SE)						
Fixed effects							
Intercept	0.72(.022)***	3.74(.14)***	6.76(.38)***	2.21(.23)***	1.06(.22)***	8.96(.37)***	3.28(.75)***
Age	-0.002(.002)	-0.013(.011)	0.011(.016)	-0.042(.042)	0.006(.013)	-0.071(.020)***	-0.062(.043)
Women	-0.16(.022)***	-0.62(.14)***	-0.62(.29)*	-0.27(.35)	-0.45(.23)	0.56(.27)*	0.31(.75)
DEP	0.11(.008)***	0.18(.040)***	0.22(.064)**	0.37(.093)***	0.14(.055)*	0.17(.038)***	0.41(.077)***
ADL	0.000(.001)	0.009(.010)	0.031(.018)	0.045(.021)*	-0.008(.017)	0.070(.021)**	0.056(.033)
SE	-0.027(.006)***	-0.100(.037)**	-0.24(.10)*	-0.32(.14)*	-0.077(.092)	0.024(.094)	-0.19(.15)
CF	0.057(.008)***	0.035(.069)	0.12(.12)	0.28(.26)	0.18(.092)*	0.012(.16)	0.51(.24)*
Linear Slope	0.088(0.019)***	-0.34(.14)*	-0.57(.26)*	1.84(.23)***	0.62(.24)*	0.33(.32)	3.86(.45)***
Age	-0.002(.001)	0.002(.007)	-0.016(.015)	0.008(.015)	-0.039(.043)	0.066(.018)***	0.020(.031)
Women	-0.032(.016)*	0.18(.12)	-0.10(.22)	-0.083(.22)	0.002(.36)	-0.64(.25)**	-0.56(.47)
DEP	-0.020(.005)***	-0.12(.027)***	-0.075(.040)	-0.14(.042)**	0.069(.063)	-0.071(.032)*	-0.034(.050)
ADL	-0.001(.001)	-0.001(.008)	-0.015(.012)	0.021(.012)	0.059(.019)**	-0.030(.016)	0.048(.021)*
SE	0.010(.004)*	0.059(.027)*	0.13(.069)	0.087(.090)	-0.19(.067)**	-0.12(.088)	0.037(.16)
CF	0.007(.006)	0.017(.049)	-0.029(.061)	0.010(.11)	-0.12(.18)	-0.038(.14)	-0.22(.16)
Quad Slope	0.002(0.002)	0.029(.015)	0.031(.035)	-0.18(.027)***	0.050(.041)	-0.064(.048)	-0.62(.075)***
Age	0.000(.000)	0.000(.001)	0.002(.001)	-0.001(.001)	0.006(.005)	-0.006(.002)**	-0.006(.004)
Women	0.002(.002)	-0.020(.013)	0.012(.027)	0.031(.024)	-0.018(.054)	0.050(.026)	0.15(.079)
DEP	0.002(.000)***	0.012(.003)***	0.007(.004)	0.011(.004)**	-0.011(.009)	0.007(.004)	-0.007(.011)
ADL	0.000(.000)*	0.000(.001)	0.001(.001)	-0.003(.002)	-0.009(.004)*	0.001(.002)	-0.011(.003)**
SE	-0.001(.000)**	-0.007(.003)*	-0.007(.008)	-0.007(.007)	0.030(.009)**	0.010(.014)	0.018(.018)
CF	0.000(.001)	-0.004(.005)	0.005(.007)	-0.001(.010)	0.014(.030)	0.006(.020)	0.016(.021)
Random effect							
Intercept	–	–	–	–	–	–	–
Linear Slope	0.14(.009)***	0.14(.000)***	0.14(0.009)***	0.14(.009)***	0.14(.009)***	0.14(.009)***	0.14(.009)***
Quad Slope	0.002(.000)***	0.002(.00)***	0.002(0.000)***	0.002(.000)***	0.002(.000)***	0.002(.000)***	0.002(.000)***

*SE* Standard errors, *Women* 1, *Quad* Quadratic, *DEP* Depressive symptomatology, *ADL* Activities of daily living, *SE* Social engagement, *CF* Cognitive functioning

**Fig. 1:**
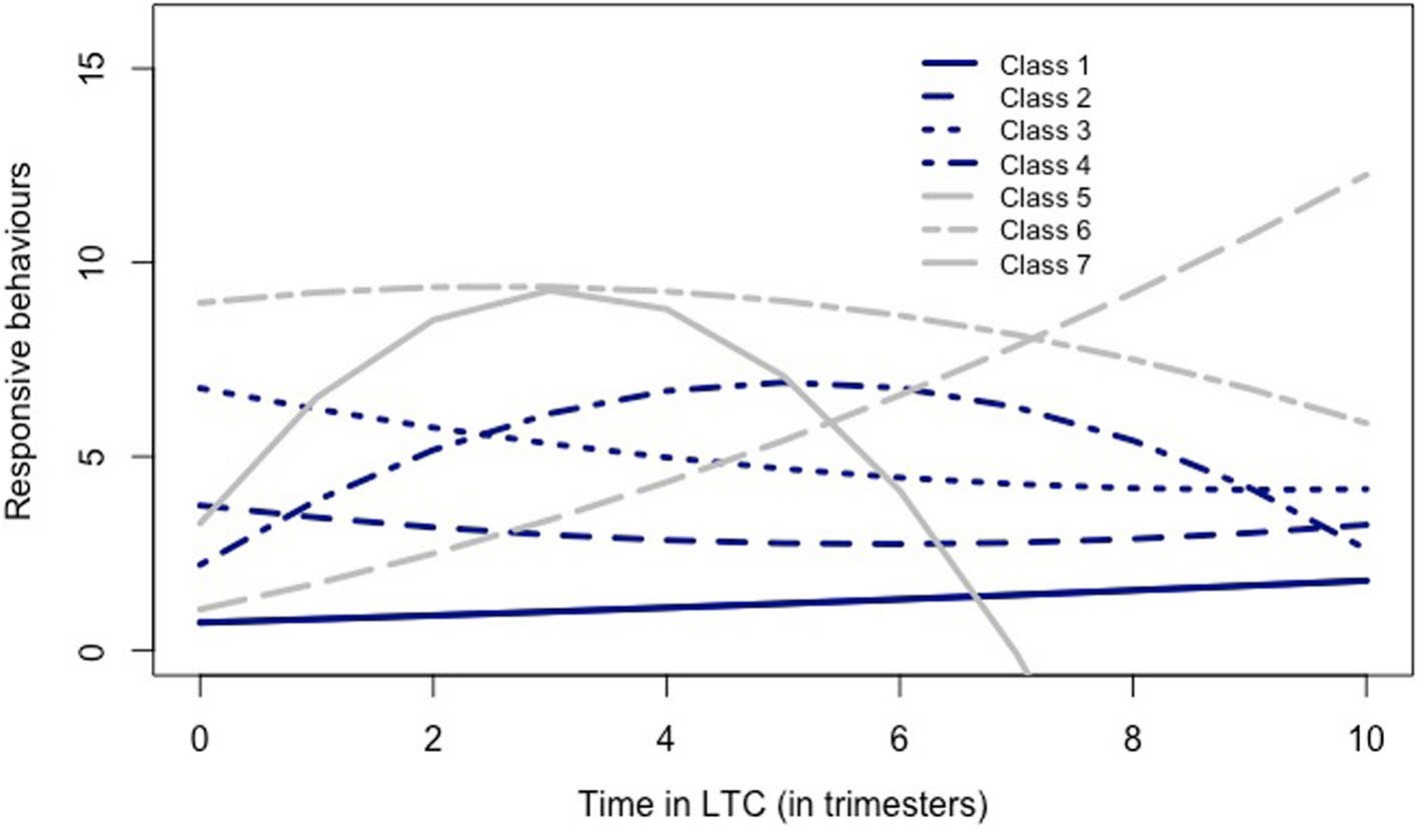
Average trajectories of responsive behaviours related to dementia for the 7 classes. Class 1: *N* = 11,601 – 69% of sample, lowest frequency/intensity; slightly increasing behaviours; class 2: *N* = 2220 – 13% of the sample, moderate frequency/intensity; slightly decreasing behaviours; class 3: *n* = 917; 5% of the sample, high frequency/intensity; decreasing behaviours; class 4: *N* = 916; 5% of the sample, low frequency/intensity; increasing followed by declining behaviours; class 5: *N* = 485; 3% of the sample, low frequency/intensity; rapidly increasing behaviours; class 6: *N* = 360; 2% of the sample, highest frequency/intensity; no change in behaviours; class 7: *N* = 301; 2% of the sample, low frequency/intensity; rapidly increasing followed by rapidly declining behaviours

Men with higher depressive symptomatology, higher cognitive impairment, and less socially engaged upon entry into LTC had a higher frequency of responsive behaviours. Women with higher depressive symptomatology demonstrated a negative change in the slope of responsive behaviour (flatter increase) over time and more social engagement was related to a positive change in the slope (steeper increase). Higher depressive symptomatology and more ADL impairment were associated with a positive quadratic change (accelerated increase) in the slope and social engagement was associated with a negative quadratic change (decelerated increase). The model estimated mean trajectory of Class 1 is depicted by a jagged solid line in Fig. 1.

#### Class 2

The second largest class was composed of 2220 individuals (13% of the sample) who presented a low frequency of responsive behaviours upon entry in LTC (3.74; SE = 0.14) and that decreased at a linear rate of -0.34 (SE = 0.14). Men with higher depressive symptomatology and lower levels of social engagement upon entry in LTC had a higher frequency of responsive behaviours. Having higher depressive symptomatology and less social engagement was associated with a negative change in the slope of responsive behaviour over time (steeper decrease). Higher depressive symptomatology was associated with a positive quadratic change in the slope (levelling off of the decrease) and social engagement was associated with a negative quadratic change (accelerated decrease; see Table 4). See the dashed line in Fig. 1.

#### Class 3

The third largest class was composed of 917 individuals (5% of the sample) who presented a higher frequency of responsive behaviours upon entry in LTC (6.76; SE = 0.38) that decreased at a linear rate of -0.57 (SE = 0.26). These individuals started with more behaviours at entry but a faster rate of decline in behaviours than those in class 2. Men with higher depressive symptomatology and lower levels of social engagement upon entry into LTC had a higher frequency of responsive behaviours. None of the covariates were statistically significantly associated with the linear or quadratic slopes (see Table 4). See the dotted line in Fig. 1.

#### Class* 4*

The fourth class was composed of 916 individuals (5% of the sample) who presented a low frequency of responsive behaviours upon entry in LTC (2.21; SE = 0.23) and that increased at a linear rate of 1.84 (SE = 0.23) after entering LTC followed by a levelling off in the later months (negative quadratic trend) at a rate of -0.18 (SE = 0.03). Higher depressive symptomatology, more ADL impairment, and lower levels of social engagement upon entry in LTC was associated with a higher frequency of responsive behaviours. Having a higher level of depressive symptomatology was associated with a negative change in the slope of responsive behaviour over time (flatter increase). Higher depressive symptomatology was associated with a positive quadratic change in the slope (see Table 4). See the dot dash line in Fig. 1.

#### Class* 5*

The fifth class was composed of 485 individuals (3% of the sample) who presented a low frequency of responsive behaviours upon entry into LTC (1.06; SE = 0.22) and that increased at a linear rate of 0.62 (SE = 0.24) after entering LTC. Higher depressive symptomatology and cognitive impairment upon entry into LTC were associated with a higher frequency of responsive behaviours. Having more ADL impairment and less social engagement was associated with a steeper increase in the slope of responsive behaviour over time. Higher social engagement was associated with a positive quadratic change in the slope and more ADL impairment was associated with a negative quadratic change in the slope (see Table 4). See the long dashed line in Fig. 1.

#### Class* 6*

The sixth class was composed of 360 individuals (2% of the sample) who presented a high frequency of responsive behaviours upon entry in LTC (8.96; SE = 0.37) and that remained stable (no significant change in the slope) over the course of their stay in LTC. People living with dementia who were younger and had a higher level of depressive symptomatology and more impairment in ADL upon entry in LTC were more likely to exhibit a higher frequency of responsive behaviours. Younger women with more depressive symptomatology showed a negative change in the slope (decreasing slope). Older age was associated with a negative quadratic change in the slope (accelerated decrease, see Table 4). See the two dash line in Fig. 1.

#### Class* 7*

The seventh class was composed of 305 individuals (2% of the sample) who presented a low frequency of responsive behaviours upon entry in LTC 3.28 (SE = 0.75) and that increased at a linear rate of 3.86 (SE = 0.45) after entering LTC followed by a levelling off in the later months (negative quadratic trend) at a rate of -0.62 (SE = 0.075). Having a higher level of depressive symptomatology and more cognitive impairment upon entry into LTC was associated with a higher frequency of responsive behaviours. Having more impairment with ADL was associated with a positive change in the slope (steeper increase in the slope) followed by a negative quadratic change in the slope (levelling off as time passes; see Table 4). See the solid line in Fig. 1.

### Markers of class membership

Cognitive functioning, social engagement, depressive symptomatology and ADL were markers of class membership. Results indicate that the odds of being in classes 2 (Estimate = 0.27***; SE = 0.021), 3 (Estimate = 0.33***; SE = 0.03), 4 (Estimate = 0.26***; SE = 0.04), 5 (Estimate = 0.23***; SE = 0.04), 6 (Estimate = 0.42***; SE = 0.03), and 7 (Estimate = 0.21***; SE = 0.03) compared to class 1 increased for individuals with higher depressive symptomatology. The odds of being in classes 2 (Estimate = -0.11***; SE = 0.03), 3 (Estimate = -0.19**; SE = 0.06), 5 (Estimate = -0.17**; SE = 0.06), 6 (Estimate = -0.27***; SE = 0.07), and 7 compared to class 1 increased for those with less social engagement. The odd of being in classes 2 (Estimate = 0.38***; SE = 0.04), 3 (Estimate = 0.42***; SE = 0.05), 4 (Estimate = 0.36***; SE = 0.07), 6 (Estimate = 0.59***; SE = 0.09), and 7 (Estimate = 0.34***; SE = 0.07) compared to class 1 increased for individuals with more cognitive impairment. The odds of being in class 4 (Estimate = -0.03**; SE = 0.01) and class 7 (Estimate = -0.03*; SE = 0.01) compared to class 1 increased for individuals with less impairment with ADL.

## Discussion

Our results demonstrate the existence of heterogeneity in trajectories of responsive behaviours for people living with dementia in LTC that is best represented by seven classes of trajectories rather than one homogenous group. This paper extends our previous work on the trajectory of dementia-related responsive behaviours and is the first paper to identify classes of trajectories of these behaviours in LTC. These findings have important implications for how we plan for the integration of residents into LTC homes. Our study provides more information about the factors that are associated with individuals who are likely to be presenting more behaviours.

Our results demonstrate that the majority (69%) of people living with dementia entering LTC present few responsive behaviours and that these behaviours increase gradually over time (i.e., class 1). This class resembles the results found from our previous study which examined the trajectory of older adults after they transition to LTC [23]. Unlike our previous publication, this study further unravels the distinct trajectories that older adults demonstrate as they continue to live in LTC, highlighting how some factors can put individuals at an increased risk of experiencing changes in behaviours and how some individuals might not adapt as well as others when moving to LTC.

People living with dementia in classes four and seven appear to be those most affected by the transition to LTC. It represents the individuals who enter LTC with few responsive behaviours but who exhibit a steep increase in behaviours right after entering LTC followed by a steep decrease in behaviours in later months. Individuals in these two classes are more likely to have higher depressive symptomatology and more cognitive impairment and less impairment on ADL than those in class one. These are the only two classes predicted by ADL. One possible explanation is that individuals doing better on ADL might find the initial transition to LTC more difficult especially if they feel they are still capable of living at home independently. Given their higher level of functioning, these individuals also have more of the physical capabilities needed to exhibit physical behaviours related to dementia. These individuals would benefit most from interventions aimed at easing people’s transition into LTC and from more person-centred care approaches by getting to know the individual before they enter LTC. A systematic review paper found that interventions aimed at increasing person-centred care approaches were successful in decreasing behaviours related to dementia in LTC [41]. This entails increasing training for LTC staff so that they see the people living with dementia as individuals and instilling the importance of communication between LTC staff, the people living with dementia and their families to better understand their likes, dislikes and needs. Special attention should also be directed towards those who are still functioning well independently to make sure their transition is as smooth as possible.

Our results also identified a class of older adults (class 5) who present few behaviours upon entry in LTC but who demonstrate a linear increase over time; one that is more pronounced than those reported in class 1. These individuals will continue to demonstrate an increase in behaviours over time unless efforts are made to address the cause of these behaviours. Compared to individuals in class 1, individuals in class 5 demonstrate more depressive symptomatology and more cognitive impairment highlighting the importance of depression prevention and treatment. Compared to those within the class, those with more impairment with ADL and less social engagement are likely to show a steeper increase in behaviours suggesting that the rate of change in behaviours could be haltered with more opportunities for social engagement and initiatives for improving ADL functioning.

There is also a class of people living with dementia (class 6) who present the highest frequency of responsive behaviours upon entry in LTC and who continue to present a consistently high frequency of behaviours over time. Compared to those in class 1, those in class 6 demonstrate higher depressive symptomatology, less social engagement, and more cognitive impairment, which further reinforces the importance of depression screening and treatment and programs that enhance opportunities for participation in social activities. Class 6 is the only class where age was found as a significant predictor with older age associated with fewer behaviours. This finding aligns with other studies that have also reported a negative relationship between age and responsive behaviours [42, 43]. One possible explanation is that this class represents individuals with frontotemporal dementia. The prevalence of frontotemporal dementia varies depending on the targeted age group with some studies suggesting an average prevalence of 2.7% for those aged 65 years and older and 10% for those under 65 years of age [44]. Given our focus on those 65 years of age and older, this aligns with our reported class size (i.e., 2% of the sample). Unfortunately, we do not have data on the type of dementia making it impossible for us to verify whether those in that class are in fact individuals living with frontotemporal dementia. Frontotemporal dementia tends to occur at a younger age and is generally characterized by more behavioural changes than other types of dementia [45]. Given that most individuals in LTC are in their later years of life, younger individuals might have fewer activities that are appropriate for their age range. More research is needed that focuses specifically on individuals with frontotemporal dementia and that examines the role of interventions (e.g., a program that offers age-appropriate activities) tailored specifically to this group.

As demonstrated, depressive symptomatology, social engagement, ADL, and cognitive functioning are important in predicting class membership. This is valuable information that can be used to identify individuals at increased risk of being in classes with more responsive behaviours related to dementia. It also means that initiatives put forth in the community, while people are still living at home, could help to reduce the number of people in the classes exhibiting more behaviours. Once individuals are in LTC, actions can also be taken to modify an individual’s trajectory of responsive behaviours.

Among all the included covariates, depressive symptomatology stands out as the most consistently associated with the trajectories of responsive behaviours. Depressive symptomatology is related to the frequency of behaviours upon entry into LTC in all classes and to the linear rate of change in four classes. This is not surprising given the high prevalence of affective symptoms including depression, anxiety, and apathy across all stages of dementia [46]. Affective symptoms are also amongst the most frequent and clinically relevant behavioral and psychological symptoms (BPSD) related to dementia [47]. This further highlights the importance in assessing affective symptoms including depressive symptomatology and in implementing effective mental health interventions [23].

Social engagement also appears to play an important role in most trajectories of responsive behaviours. The longitudinal importance of social engagement further reinforces the importance of keeping people living with dementia in LTC engaged in activities with regular human contact [2]. The importance of tackling loneliness in older adults in LTC is something that can never be underestimated.

The large sample size of people living with dementia followed over numerous follow-up occasions and the state-of-the-art techniques for longitudinal analysis are clear advantages of this study. Still, some limitations are important to mention. First, because we are using observational data, the current study does not make any claims of causality. Secondly, some issues have been identified with data quality and some LTC home staff appear better equipped to collect quality data than others. Still, numerous CCRS data quality checks are in place to reduce error and CIHI reports high-quality data in their data quality documentation. Thirdly, the ABS [27] is a validated measure meant to be used as a composite score that focuses on the frequency and intensity of behaviours rather than on the different types of behaviours. Future research should explore changes in the different types of behaviours (e.g., verbal expressions, physical behaviours, socially inappropriate behaviours) and behaviour-specific risk factors (e.g., type of dementia, gender). Fourthly, antipsychotic medications are frequently used to manage behaviours related to dementia [48] and could have an impact on class membership. Unfortunately, given the complexity of the models (i.e., GMM models), and the changing nature of antipsychotic medication (time-varying covariates) use over time, it was decided not to include medication use in the models. Lastly, some factors which have been found to influence responsive behaviours such as the physical environment of the LTC home (size of the rooms and layout), the number of staff, and culture were not available in the data and therefore could not be included in our models even though these have been found as important by other researchers [21]. More research should also examine the impact of different social facets (e.g., social network size, tangible support, emotional support, and loneliness) on responsive behaviours in LTC given that only social engagement was included in the CIHI data. Given that the majority of studies are cross-sectional, further longitudinal data is needed that examines elements that were not addressed in this paper [20]. More research linking data sources is also needed to move knowledge forward and address the gaps in existing data (e.g., [49]).

More collaborative efforts [50], using several longitudinal studies, are needed to examine whether findings can be generalizable across different countries, states\provinces, and cohorts. By using consistent analytical procedures across all studies, we would be better able to explore study-specific explanations for variations in results such as differences in LTC culture across countries.

As demonstrated by the seven classes identified in this study, people living with dementia in LTC do not all experience the same trajectory of behaviours related to dementia. Groups of individuals have distinct trajectories that are associated with different biopsychosocial factors. Unlike the previous research which treated change in behaviours as a homogeneous process, this approach has allowed us to identify those at increased risk of exhibiting profiles with more changes in behaviours. The CCRS is completed for all individuals when they move into a LTC home. This information, in combination with information provided by the residents and their family members, can provide LTC staff with some guidance on how they might adjust to the LTC setting and how behaviours are likely to change in frequency and intensity over time. This information can be used to inform the care and support they receive. Based on the results of this study, interventions aimed at reducing depression and increasing social participation should be evaluated for their impact on reducing responsive behaviours. Changes in responsive behaviours and risk factors can also be monitored over time to see whether interventions are making a difference for these individuals. This study also allows us to see how, in the current context of LTC, residents may or may not adapt to their new environments over time.

## Data Availability

The data that support the findings of this study are available from the Canadian Institute for Health Information (CIHI), but restrictions apply to the availability of these data, which were used under license for the current study, and so are not publicly available. CIHI undertakes a rigorous multi-step review and authorization process of all data requests. This process was undertaken for the current study. Data are however available from CIHI (https://www.cihi.ca/en/access-data-and-reports/data-holdings/make-a-data-request) upon reasonable request and after completing all data request requirements. For further information about requesting the data from this study contact AR (email: arobitai@uottawa.ca).
